# Heme Binding Proteins of *Bartonella henselae* Are Required when Undergoing Oxidative Stress During Cell and Flea Invasion

**DOI:** 10.1371/journal.pone.0048408

**Published:** 2012-10-29

**Authors:** MaFeng Liu, Yann Ferrandez, Emilie Bouhsira, Martine Monteil, Michel Franc, Henri-Jean Boulouis, Francis Biville

**Affiliations:** 1 UMR BIPAR Université Paris-Est, Ecole nationale vétérinaire d’Alfort, INRA-Anses-UPEC-ENVA, Maisons-Alfort, France; 2 Key Laboratory of Zoonosis, Ministry of Education, Institute of Zoonosis, Jilin University, Changchun, People’s Republic of China; 3 Laboratoire de parasitologie, Université de Toulouse, INP, ENVT, Toulouse, France; 4 Département de Microbiologie, Institut Pasteur, Paris, France; Charité-University Medicine Berlin, Germany

## Abstract

*Bartonella* are hemotropic bacteria responsible for emerging zoonoses. These heme auxotroph alphaproteobacteria must import heme for their growth, since they cannot synthesize it. To import exogenous heme, *Bartonella* genomes encode for a complete heme uptake system enabling transportation of this compound into the cytoplasm and degrading it to release iron. In addition, these bacteria encode for four or five outer membrane heme binding proteins (Hbps). The structural genes of these highly homologous proteins are expressed differently depending on oxygen, temperature and heme concentrations. These proteins were hypothesized as being involved in various cellular processes according to their ability to bind heme and their regulation profile. In this report, we investigated the roles of the four Hbps of *Bartonella henselae,* responsible for cat scratch disease. We show that Hbps can bind heme *in vitro*. They are able to enhance the efficiency of heme uptake when co-expressed with a heme transporter in *Escherichia coli*. Using *B. henselae* Hbp knockdown mutants, we show that these proteins are involved in defense against the oxidative stress, colonization of human endothelial cell and survival in the flea.

## Introduction

*B. henselae* is now recognized as one of the most common zoonoses acquired from animal companions in industrialized countries [1]. The bacterium causes cat scratch disease as well as a number of other syndromes associated with tumoral proliferation of endothelial cells [2]. Most *Bartonellae* species appear to share a similar natural cycle that involves arthropod transmission, followed by exploitation of a mammalian host. Each *Bartonella* species appears to be highly adapted to one or several reservoir hosts in which it causes long-lasting intra-erythrocytic bacteremia as a hallmark of infection. Before colonizing erythrocytes, the bacteria need to replicate and become competent in a primary niche [3] such as endothelial cells, although other nucleated cells might constitute part of the primary niche [4]. Bacterial persistence in erythrocytes is an original strategy, and is considered an adaptation to the mode of transmission by bloodsucking arthropod vectors. The invasion of erythrocytes might also be a strategy for *Bartonellae* species to obtain heme, an absolute requirement for growth [5]. Not all of the already sequenced *Bartonellae* genomes contain heme biosynthesis genes [6]. Moreover, these genomes do not encode for siderophore biosynthesis or a complete iron Fe^3+^ transport system. Only genes sharing strong homology with all compounds of an Fe^2+^ transport system, already characterized in *Yersinia pestis* [7] and *Photorhabdus luminescens* [8], are present in *Bartonellae* genomes. Moreover, *Bartonellae* genomes encode for a complete heme transport system shown to be active in *Bartonella quintana* [9].

Analysis of *Bartonellae* genomes sequenced to date clearly shows the absence of numerous genes proven to be required for *E. coli* in order to face oxidative stress [10]. Genes coding for polypeptides involved in degradation of hydrogen peroxide, like catalase and peroxidase [11], methyl sufoxide reduction (MsrA and MsrB) [12] and oxidative stress response regulation (SoxR, OxyR) [13], [14] are not contained in *Bartonellae* genomes. Based on the above information relating to *Bartonellae* genome contents, it can be hypothesized that *Bartonellae* are highly sensitive to oxidative stress. However, hydrogen peroxide challenges performed with *B. bacilliformis* clearly show that this bacterium can efficiently face exposure to 1 mM H_2_O_2_ for 30 min [15]. Moreover, the lifestyle of *Bartonellae* suggests that these bacteria must face oxidative stress after the blood meal of the arthropod vector [16]. *Bartonellae* genomes are small in size, 1.2 to 2 M bases. In spite of their reduced size, they encode for three to five heme binding proteins (Hbps) [17]. Comparison of *B. quintana* Hbps (HbpA, HbpB, HbpC and HbpD) shows that three of these proteins are similar in size and peptide sequences. The HbpA, HbpC and HbpD sequences of these approximately 30 kDa polypeptides are nearly 53% identical. HbpB can be distinguished from other Hbps, as this polypeptide is about 11 kDa bigger than the other Hbps and contains a central part not present in other Hbps from *B. quintana* [17].

To survive and multiply, *Bartonellae* are forced to encounter various environments in their hosts and vectors. In mammals, free heme is rare [18] and the blood oxygen concentration is low (5% compared to 21% in the atmosphere) [19]. In contrast, in arthropod vectors, toxic heme level is found in the gut and the oxygen concentration is high [16]. The regulation pattern of *hbp* gene expression was thus investigated in *B. quintana* [19]. Under all conditions tested, *hbpA* was more strongly expressed than other *hbp* genes [19]. Based on their regulatory pattern, *hbp* genes were divided into two groups. The first contained *hbpB* and *hbpC,* over expressed under conditions that mimick the gut arthropod environment (high heme concentration and low temperature, high O_2_ concentration). The transcription level of *hbpB* and *hbpC* was higher at high heme concentrations (2.5 to 5 mM) [19]. Those authors suggested that HbpB and HbpC play a critical role in the arthropod gut [19]. The transcription of *hbpA, hbpD* and *hbpE* was increased at low heme concentrations (0.05 mM) at 37°C. The authors suggested that HbpA, HbpD and HbpE are required when the free heme concentration is low, such as in blood circulation in the mammalian host. However, it was recently shown that transcription of *B. henselae hbpA* is also significantly increased at 28°C, suggesting that HbpA could protect *B. henselae* from heme toxicity in the arthropod gut [20].

The function of HbpA was first investigated in *B. henselae*. Controversial results excluded a direct role for HbpA in the heme uptake process [21], [22]. Various reports demonstrated that HbpA of *B. henselae*, which shares homology with Opa from *Neisseria meningitidis* [23], plays a role in the endothelial cell adhesion process [24], [25]. HbpB of *B. tribocorum* was shown to be required for establishing long-term bacteremia in a rat model, but its role has not been elucidated [26]. HpbC of *B. henselae* was recently identified as a heme detoxifying protein [20]. Finally, it was suggested that HbpD of *B. henselae* is required for survival in endothelial cells [27].

In this report, we investigated the activity of four Hbps (HbpA, B, C, D) of *B. henselae* using both homologous and heterologous (*E. coli*) models. In *E. coli*, the ability to bind Congo red and heme was investigated *in vivo* and *in vitro* respectively. In *B. henselae*, *hbp* knockdowns were checked for their growth ability, oxidative stress response and capacity to invade and survive within endothelial cells. Moreover, these mutants were tested for their ability to develop within the *B. henselae* arthropod vector *Ctenocephalides felis*.

## Materials and Methods

### Ethics Statement

Animals were handled in strict accordance with good animal practice as defined by the relevant European (European standards of welfare for animals in research), and/or local animal welfare bodies. Animal work performed at the Ecole Nationale vétérinaire d’Alfort (ENVA/ANSES) was reviewed and approved by the institute’s ethics committee on September 2011(agreement n°:14/06/2011-1). Animal work performed at the Ecole nationale vétérinaire de Toulouse was reviewed and approved by the INRA Toulouse/ENVT ethics committees (agreement n°: MP/01/22/06/09) for 3 years.

### Bacterial Strains and Plasmids

Bacterial strains and plasmids used in this study are listed in Table 1.

**Table 1:** Strains and plasmids used in this study.

E. coli strains	Genotype	Source or reference
XL1-Blue	F^−^ supE44 hdsR17 recA1 endA1 gyrA46 thi relA1 lac^−^ F′ proAB^−^ lacI^q^lacZΔM15 Tn10 (Tet^R^)	Laboratory collection
FB8.27	FB8 Δlac entF::TnphoA′5, Tet^R^	[44]
XL1-Blue pBAD24	XL1-Blue carrying pBAD24, Amp^R^	This study
XL1-Blue pBAD24::hbpA	XL1-Blue carrying pBAD24::hbpA, Amp^R^	This study
XL1-Blue pBAD24::hbpB	XL1-Blue carrying pBAD24::hbpB, Amp^R^	This study
XL1-Blue pBAD24::hbpC	XL1-Blue carrying pBAD24::hbpC, Amp^R^	This study
XL1-Blue pBAD24::hbpD	XL1-Blue carrying pBAD24::hbpD, Amp^R^	This study
FB8.27 pBAD24	FB8.27 pBAD24, Tet^R^, Amp^R^	This study
FB8.27 pBAD24::hbpA	FB8.27 pBAD24::hbpA, Tet^R^, Amp^R^	This study
FB8.27 pBAD24::hbpB	FB8.27 pBAD24::hbpB, Tet^R^, Amp^R^	This study
FB8.27 pBAD24::hbpC	FB8.27 pBAD24::hbpC, Tet^R^, Amp^R^	This study
FB8.27 pAM239::hemR, pBAD24	FB8.27 pAM239::hemR, pBAD24, Tet^R^, Spc^R^, Amp^R^	This study
FB8.27 pAM239::hemR, pBAD24::hbpA	FB8.27 pAM239::hemR, pBAD24::hbpA, Tet^R^, Spc^R^, Amp^R^	This study
FB8.27 pAM239::hemR, pBAD24::hbpB	FB8.27 pAM239::hemR, pBAD24::hbpB, Tet^R^, Spc^R^, Amp^R^	This study
FB8.27 pAM239::hemR, pBAD24::hbpC	FB8.27 pAM239::hemR, pBAD24::hbpC, Tet^R^, Spc^R^, Amp^R^	This study
FB8.27 pBAD24::hbpC	FB8.27 pBAD24::hbpC, Tet^R^, Amp^R^	This study
FB8.27 pAM239::hemR, pBAD24::hbpD	FB8.27 pAM239::hemR, pBAD24::hbpD, Tet^R^, Spc^R^, Amp^R^	This study
Bartonella strains	Genotype	Source or reference
B. henselae Houston-1	Houston-1, ATCC 49882^T^	Laboratory collection
B. henselae pNS2Trc	B. henselae carrying pNS2Trc, Amp^R^	This study
B. henselae pNS2Trc::hbpA _AS_	B. henselae carrying pNS2Trc::hbpA _AS_, Amp^R^	This study
B. henselae pNS2Trc::hbpB _AS_	B. henselae carrying pNS2Trc::hbpB _AS_, Amp^R^	This study
B. henselae pNS2Trc::hbpC _AS_	B. henselae carrying pNS2Trc::hbpC _AS_, Amp^R^	This study
B. henselae pNS2Trc::hbpD _AS_	B. henselae carrying pNS2Trc::hbpD _AS_, Amp^R^	This study
Plasmids		
pBAD24	pBR322 araC, arabinose-inducible promoter, Amp^R^	Laboratory collection
pNS2Trc	Km^R^	[49]
pAM239::hemR	pAM239 carrying hemR from Serratia marcesns	[74]
pBAD24::hbpA	pBAD24 carrying hbpA from B. henselae, Amp^R^	This study
pBAD24::hbpB	pBAD24 carrying hbpB from B. henselae, Amp^R^	This study
pBAD24::hbpC	pBAD24 carrying hbpC from B. henselae, Amp^R^	This study
pBAD24::hbpD	pBAD24 carrying hbpD from B. henselae, Amp^R^	This study
pNS2Trc::hbpA _AS_	pNS2Trc carrying anti-sense hbpA, km^R^	This study
pNS2Trc::hbpB _AS_	pNS2Trc carrying anti-sense hbpB, km^R^	This study
pNS2Trc::hbpC _AS_	pNS2Trc carrying anti-sense hbpC, km^R^	This study
pNS2Trc::hbpC _AS_	pNS2Trc carrying anti-sense hbpC, km^R^	This study

### Media and Growth Conditions

Bovine hemoglobin (Hb) and 2, 2′dipyridyl (Dip) were obtained from Sigma-Aldrich France (38297 Saint-Quentin FallavierFrance). Heme was dissolved immediately before use in 0.02 M NaOH. Hb was dissolved in 100 mM NaCl. Heme and Hb solutions were filter-sterilized with 0.20 µm Millipore filters for bacterial growth experiments. *E*. *coli* strains were grown on LB medium (Sigma), M63 minimal medium, aerobically at 37°C [28] or on Congo red plates. M63 medium was supplemented with 0.4% glycerol (Gly) as carbon source. Solid media and soft agar respectively contained 1.5% or 0.7% Difco agar. Congo red plates consisted of solid BHI medium (Difco) supplemented with Congo red dye (0.02% final concentration). Iron-depleted medium was obtained with the addition of Dip at an 80 µM final concentration. Antibiotics were added to the following final concentrations (µg ml^−1^): ampicillin (Amp), 50; kanamycin (Km), 50; and spectinomycin (Spc), 50. Arabinose (Ara) was added to a final concentration of 0.02%, 0.2% or 0.4% for induction of the P_ara_ promoter. *B. henselae* was grown on a Columbia blood agar (CBA) plate containing 5% defibrinated sheep blood (Biomérieux; ref 43041; 5, rue des Aqueducs BP 10 - 69290 Craponne, France) or in Schneider’s medium (Gibco; Route de L’Orme des Merisiers Immeuble Discovery - Zone Technologique 91190 Saint Aubin, France) supplemented with 10% fetal calf serum [29] at 35°C under a 5% CO_2_ atmosphere. For flea infection assays, *Bartonella* strains were collected after 5 days of growth on CBA plates and suspended in PBS buffer. The bacterial suspension was diluted with PBS to obtain a cell density of 1.98×10^8^ bacteria/ml. The survival of bacteria in PBS buffer was not significantly decreased after 24 h storage at room temperature.

### Congo Red Binding Assay

Tested strains containing pBAD derivatives expressing Hbps were grown overnight at 37°C in LB medium containing 50 µg/ml ampicillin. Two ml of LB medium containing 50 µg/ml ampicillin were inoculated to an OD_600_ of 0.05 with overnight culture and grown at 37°C. Expression was induced at an OD_600_ of about 0.6 for 2 h by adding arabinose (0.4% final concentration). Cultures were diluted, plated on Congo red plates and incubated at 37°C for 24 h.

### *E. coli* Heme-dependent Growth Assays

Tested strains were grown for 18 h in M63 medium without iron with 0.4% glycerol as carbon source, and in the presence of 0.02% arabinose. Cultures were checked for OD at 600_nm_ and adjusted to OD_600_ = 1. A 100 µl sample of the bacterial suspension was mixed with 4 ml of soft agar. The mixture was poured onto M63 plates containing 0.4% glycerol, 0.02% arabinose and 80 µM Dip (M63D). Wells (5 mm in diameter) were cut in the agar and filled with 100 µl of 50 µM, 10 µM, 5 µM, or 1 µM of filter-sterilized Hb solution. Growth around the wells was recorded after 2-day incubation at 37°C. All experiments were performed in triplicate.

### Physiological Characterization of *hbp* Knockdown Strains

To evaluate the effect of *hbp* knockdown on growth of *B. henselae*, tested strains were grown both in liquid Schneider’s medium and on CBA plates. *B. henselae* pNS2Trc and *B. henselae* pNS2Trc*::hbps*_AS_ were collected after 5 days of growth on CBA plates and suspended in Schneider’s medium or phosphate buffered saline (PBS). For growth in Schneider’s medium, the OD_600_ of the bacterial suspension was adjusted to 0.05. Five ml samples of this suspension were poured into 6-well plates and grown at 35°C in the presence of 5% CO_2_. OD_600_ was checked at days 2, 4, 5 and 7 after inoculation. For growth on CBA plates, serial dilutions of bacterial suspension in PBS were plated on CBA plates and colony size was evaluated after 6 and 10 days of growth at 35°C in the presence of 5% CO_2_. All experiments were performed in triplicate.

### H_2_O_2_ Challenge

*B. henselae* pNS2Trc and *B. henselae* pNS2Trc*::hbps*_AS_ were grown on CBA plates for five days at 35°C under a 5% CO_2_ atmosphere. Bacteria collected from one plate were suspended and washed twice in PBS buffer. The cell suspension was then diluted to OD_600_ 0.5. Before H_2_O_2_ challenge, several dilutions of the tested cell suspension were spread on CBA plates (T_0_). For the challenge assay, bacteria were incubated 30 min in PBS buffer in the presence of 1 mM and 10 mM H_2_O_2_ at 35°C under a 5% CO_2_ atmosphere. After exposure to H_2_O_2_, bacteria were washed twice in PBS buffer and several dilutions plated onto CBA plates (T_1_). After 15-day incubation at 35°C under a 5% CO_2_ atmosphere, colonies were counted. Survival rate was expressed by (T_1_/T_0_) X100%. All experiments were performed in triplicate.

### Endothelial Cell Culture and Invasion Assay

Endothelial cell line Ea.hy 926 resulting from a fusion of HUVEC and lung carcinoma cell line A549 (ATCC; reference CRL 2922) were cultured in DMEM medium (Gibco) supplemented with 10% fetal bovine serum decomplemented by heating 30 min at 56°C before use. When required, kanamycin was added at a 50 µg/ml final concentration. Cells were incubated at 37°C in humidified 5% CO_2_ and cultured every 7 days using 0.025% trypsin and 1 mM EDTA in Hanks’ balanced salt solution [30]. Endothelial cells were seeded in 24-well plates at a density of 10^4^ cells/well. After 6 days, cell number was estimated as 1.5×10^5^/well. *Bartonella* strains harvested from CBA plates after 5-day growth at 35°C under a 5% CO_2_ atmosphere were washed twice in modified DMEM buffer and then resuspended in the same medium. Bacterial number was adjusted to 3×10^4^/ml (1OD was estimated at 6.6×10^8^/ml). For cell invasion assays, medium in the well was removed and 0.5 ml of modified DMEM containing 1.5×10^4^ colony-forming units (CFUs) of the *B. henselae* tested strains were added to the well to obtain 0.1 multiplicity of infection (m.o.i). A low m.o.i. was used since we considered a cell excess as more suitable to visualize the full invading ability of bacteria. After remaining for 1 h at 37°C, 1.5 ml of modified DMEM medium was supplemented in the well. The number of bacteria was controlled by plating several dilutions on CBA plates, and CFUs were determined after 15 days of growth (T_0_). Mixtures were incubated at 37°C at 5% CO_2_ for 24 h. After 24 h, the number of viable bacteria was determined by plating serial dilutions of mixtures on CBA plates, and CFUs were determined after 15 days growth (T_24_). After 24 h, bacterial viability was nearly 100%. The intracellular bacterial population was quantified by the gentamicin protection assay as described by Mehock [31]. Briefly, DMEM with gentamicin (final concentration 250 µg/ml) was added to the mixture assay and removed after 2 h at 37°C in the presence of 5% CO_2_. A control *B. henselae* bacterial suspension showed no survival after 2 h exposure to gentamicin (250 µg/ml). After removing modified DMEM medium containing gentamicin, endothelial cells were then washed three times with modified DMEM medium to remove residual antibiotic. Endothelial cells were then collected after 4 min incubation with 400 µl trypsin at 37°C. After centrifuging at 12,000 rpm, cells were suspended in 1 ml of sterile water and disrupted using a 1 ml syringe and a 0.4 mm×20 mm needle and 5 pushes [32]. Microscopic controls revealed that, after 5 pushes, all cells were lysed. Cell lysates were supplemented with 100 µl 10×PBS to overcome osmotic lysis. The number of viable bacteria was determined by plating lysates on CBA plates. After 15 days of incubation at 35°C under a 5% CO_2_ atmosphere, colonies were counted (T_I_). The invasion rate was expressed as (T_I_/T_0_)x100%. Each assay was performed in double wells and all experiments were performed in triplicate.

### Survival Assay in Endothelial Cells

To perform survival assays of *B. henselae* pNS2Trc and *B. henselae* pNS2Trc*::hbps_AS_* in endothelial cells, the monolayer contained in mixtures challenged for gentamicin killing was washed three times and incubated in modified DMEM medium at 37°C and 5% CO_2_ for 24 and 48 h. Cells were treated as described above and the number of viable bacteria was determined by plating the lysates on CBA plates. After 15-day incubation at 35°C under a 5% CO_2_ atmosphere, colonies were counted (T_S24_ or T_S48_). The survival rate was expressed as (T_S24_/T_I_ or T_S48_/T_I_)x100%. Each assay was performed in double wells and all experiments were performed in triplicate.

### Flea Maintenance and Supply

Strain *Ctenocephalides felis* (*C. felis*) (*Siphonaptera: Pulicidae*) originating from a wild strain harvested from a cat has been maintained on cat under laboratory conditions since 1990. A new generation is obtained every 2 to 4 weeks. Fleas were controlled to be PCR negative for *B. henselae* using primers bh2390fo, and bh2390re (Table 2).

**Table 2:** Primers used in this study.

Primer	Gene	Organism	Sequence
**HbpABhamont**	*hbpA*	*B. henselae*	5′ CTAGCTAGCAGGAGGAATTCACCATGAATATAAAATCTTTAATGA 3′
**HbpABhaval**	*hbpA*	*B. henselae*	5′ CGGGGTACCTCAGTGGTGGTGGTGGTGGTGGAATTTGTACGCTACACCAACACGG 3′
**HbpBBhamont**	*hbpB*	*B. henselae*	5′ CTAGCTAGCAGGAGGAATTCACCATGAATACGAAACGTTTAATAACAG 3′
**HbpBBhaval**	*hbpB*	*B. henselae*	5′ ATCCCCGAAGCTTATGGTTTAGTGGTGGTGGTGGTGGTGGAATTTGTAAGCGACAC 3′
**HbpCBhamont**	*hbpC*	*B. henselae*	5′ CTAGCTAGCAGGAGGAATTCACCATGAAATCGCGTGTTCAAATAT3′
**HbpCBhaval**	*hbpC*	*B. henselae*	5′ CGGGGTACCTCAGTGGTGGTGGTGGTGGTGAAATTTATAAGCGACACCAACACGG 3′
**HbpDBhamont**	*hbpD*	*B. henselae*	5′ CTAGCTAGCAGGAGGAATTCACCATGACTACAAAATATTTAATCACAA 3′
**HbpDBhaval**	*hbpD*	*B. henselae*	5′ CGGGGTACCTCAGTGGTGGTGGTGGTGGTGAAACTTGTACGCTACACCAACACGA 3′
**hbpAantisensamt**	Antisens *hbpA*	*B. henselae*	5′ CCCGGATCCTTAGAATTTGTACGCTACACC3′
**hbpAantisensavl**	Antisens *hbpA*	*B. henselae*	5′CCCTCTAGAATGAATATAAAATCTTTAATG 3′
**hbpBantisensamt**	Antisens *hbpB*	*B. henselae*	5′ CCCGGATCCTTAGAATTTGTAAGCGACACC3′
**hbpBantisensavl**	Antisens *hbpB*	*B. henselae*	5′ CCCTCTAGAATGAATACGAAACGTTTAATAAC3′
**hbpCantisensamt**	Antisens *hbpC*	*B. henselae*	5′ CCCGGATCCTTAAAATTTATAAGCGACACC3′
**hbpCantisensavl**	Antisens *hbpC*	*B. henselae*	5′ CCCTCTAGAATGAATATAAAATGGTTAATA3′
**hbpDantisensamt**	Antisens *hbpD*	*B. henselae*	5′ CCCGGATCCTCAAAACTTGTACGCTACACCA 3′
**hbpDantisensavl**	Antisens *hbpD*	*B. henselae*	5′ CCCTCTAGAATGACTACAAAATATTTAATCACA 3′
**bh2390fo**	bh2390	*B. henselae*	5′-GGTGAATGTGTGCAAAGTTTTAAG 3′
**bh2390re**	bh2390	*B. henselae*	5′ CCAATAAACGCCAACAAAGAC 3′.
**Cf18Sf**	18S rDNA	*C. felis*	5′ TGCTCACCGTTTGACTTGG 3′
**Cf18Sr**	18S rDNA	*C. felis*	5′ GTTTCTCAGGCTCCCTCTCC 3′

### Feeding of *C. felis* with *B. henselae* pNS2Trc- or *B. henselae* pNS2Trc*::hbps_AS_*-infected Blood

Dog blood used in all experiments was obtained from 3 beagles (15 ml obtained from each dog) from the Ectoparasite Laboratory of the National Veterinary School in Toulouse, France. The absence of *Bartonella* spp. in the blood of these dogs was confirmed by PCR using primers bh2390fo, and bh2390re (Table 2). Lithium heparin–coated vacutainer tubes (Venosafe, Terumo Europe) were used to draw blood by venipuncture. Blood functional complement was deactivated by maintaining blood samples at room temperature for 2 h after the blood test and before storing them at 4°C. Blood samples were stored less than 48 h at 4°C. When required, kanamycin was added to blood at a 50 µg/ml final concentration. Kanamycin was previously determined to have no effect on *C. felis* feeding, viability or egg production. A total of 500 unfed fleas (males and females aged between 8–10 days) were placed in a plexiglas box in contact with a glass feeder closed at the bottom by a parafilm membrane. To stimulate flea blood-feeding, a constant temperature (38.5°C) was maintained by a water-jacket circulation system through the glass feeder. For blood infection, 500 µL of bacterial suspension at a concentration of approximately 1.98×10^8^ bacteria/ml in PBS were added to 5 ml of blood. Viability of *Bartonella* in blood was about 100% after 2-h incubation. Blood was complemented by bacterial suspension for the first two days of feeding. Then, fleas were fed with uninfected dog blood for the next 8 days. Every 24 h, the glass feeder was cleaned, a new parafilm membrane was stretched and blood was changed. At the same time, flea feces were collected. All samples were stored at −20°C until PCR analysis. Ethanol 70% was added to flea feces samples.

### Genetic Techniques

*E. coli* cells were transformed by the calcium chloride method [33]. *Bartonella* cells were transformed by electroporation as previously described [34].

### DNA Manipulations

A small-scale plasmid DNA preparation was performed using a QIAprep Spin Miniprep kit (Qiagen; QIAGEN France 3 av du Canada LP 809 91974 Courtaboeuf). Restriction, modification, and ligation were carried out according to the manufacturer’s recommendations. DNA fragments were amplified in a Hybaid PCR thermocycler using Phusion DNA polymerase (Finnzymes). Nucleotide sequencing was performed by Eurofins MWG Operon. Purification of DNA fragments from the PCR reaction, restriction reaction or agarose gels was performed using the Macherey-Nagel NucleoSpin® Extract II kit.

### Construction of a Recombinant Vector Expressing Hbps of *B. henselae*

Complete *B. henselae hbpA, hbpB, hbpC and hbpD* genes with a C-terminal Histag (6 His) were amplified by PCR from *B. henselae* chromosomal DNA using primers HbpABhamont and HbpABhaval, HbpBBhamont and HbpBBhaval, HbpCBhamont and HbpCBhaval or HbpDBhamont and HbpDBhaval, respectively (Table 2). The fragments amplified (890 bp for *hbpA*, 881 bp for *hbpC* and 875 bp for *hbpD*) were purified and digested with NheI and KpnI. The *hbpB* fragment (1235 bp) was purified and digested with NheI and Hind III. Then, fragments were ligated with pBAD24 plasmid digested with NheI and KpnI or NheI and Hind III. Ligation mixtures were introduced into CaCl_2_-competent *E. coli* strain XL1 Blue cells and transformants were selected on LB plates containing ampicillin. Clones were screened by the PCR method with corresponding primers. Six PCR-positive clones were then sequenced.

### Construction of a Knockdown Vector for Decreasing the Amount of Hbp in *B. henselae*

The entire coding region of *hbpA, hbpB, hbpC and hbpD* was amplified by PCR from the *B. henselae* chromosomal DNA using primers hbpAantisensamt and hbpAantisensavl, hbpBantisensamt and hbpBantisensavl, hbpCantisensamt and hbpCantisensavl or hbpDantisensamt and hbpDantisensavl, respectively (Table 2). The PCR product (846 bp for *hbpA*, 1,182 bp for *hbpB,* 837 bp for *hbpC* and 831 bp for *hbpD*) was purified, digested with BamHI and XbaI and then ligated with plasmid pNS2Trc digested with BamHI and XbaI. Ligation mixtures were introduced into CaCl_2_-competent *E. coli* strain XL1 Blue cells. Transformants were screened by PCR with corresponding primers. Six PCR-positive clones were then sequenced.

### Extraction of DNA from Flea Feces

DNA was extracted from flea feces using the Nucleospin tissue kit according to the manufacturer’s instructions (NucleoSpin® Tissue, Macherey-Nagel; 1, Rue Gutenberg, B. P. 135, 67722 Hoerdt France). The quantity of biological material used for DNA extraction was about 30–40 mg of feces. Flea feces were incubated overnight to 56°C for the pre-lysis step. For all samples, the final elution volume was 100 µL. The concentration of DNA extraction in all samples was measured using a nanodrop spectrophotometer (NanoDrop 2000, Thermo Scientific).

### Detection of *B. henselae* DNA from Flea Feces

DNA of *B. henselae* was detected by amplification of a 1,052 bp fragment containing *B. henselae bh02390* gene https://www.genoscope.cns.fr/agc/microscope/home/index.php using primers bh2390fo, and bh2390re (Table 2). DNA of *C. felis* was detected by amplification of a fragment of *C. felis* 18S rDNA using primers Cf18Sf and Cf18Sr (Table 2) [35]. Amplifications were performed with at least 20 ng of DNA extract for flea feces. Each reaction was conducted in a total volume of 20 µL with 0.5 µM of each primer, 200 µM of each dNTP, 4 µL of 5× PCR buffer G/C and 0.02U/µl of Taq DNA polymerase (Phusion® High-Fidelity DNA Polymerase, Thermo scientific). The PCR program was as follows: an initial denaturation step for 30 s at 98°C, followed by 40 cycles of denaturation for 10 s at 98°C, annealing for 30 s at 55°C and extension for 1 min at 72°C, and a final extension step at 72°C for 7 min.

### Expression and Purification of Recombinant His-tagged Hbps

Strain XL1-Blue pBAD24*::hbpA*, XL1-Blue pBAD24*::hbpB*, XL1-Blue pBAD24*::hbpC* and XL1-Blue pBAD24*::hbpD* were grown overnight at 37°C in LB medium containing 50 µg/ml ampicillin. Then, 200 ml of LB medium containing 50 µg/ml ampicillin was were inoculated to an OD_600_ of 0.05 with the overnight culture and grown at 37°C. Expression was induced at an OD_600_ of about 0.6 for 2 h by adding arabinose (0.4% final concentration). Bacteria were harvested by centrifugation for 10 min at 3,000 g at 4°C, and the pellet was suspended in 20 ml binding buffer (50 mM Tris-HCl, 8 M urea, 0.05% triton, pH 8.0). Lysis of bacteria was obtained by incubation at room temperature with rotation for 3 h. The suspension was then centrifuged at 13,000 g for 30 min at 4°C. The supernatant containing the soluble fraction was mixed with 200 µl of Ni-agarose beads (Qiagen) according to the manufacturer’s instructions. Purified protein was dialyzed twice against a buffer containing 50 mM Tris-HCl to eliminate any residual imidazole and urea. The protein was stable for at least one month when kept at −80°C with 20% glycerol.

### Heme Binding Assay in vitro

For heme blotting, proteins were separated on standard 12.5% SDS gels followed by electrophoretic transfer to nitrocellulose membranes according to the protocol of Vargas [36]. Briefly, samples were mixed with loading buffer to which no DTT was added and samples were not boiled before electrophoresis. 1 µg of HbpA, HbpB, HbpC and HbpD or 3 µg of BSA was separated on 12.5% SDS-PAGE. One gel was stained with Coomassie brilliant Blue R. Another gel was transferred to nitrocellulose using the general methods of Towbin *et al*. [37]. The heme binding blot was done according to the protocol of Carroll *et al.* [22] **.** Briefly, the resulting blots were rinsed with Tris-buffered saline containing 0.1% Tween 20 (TBST; 10 mM pH 8.0 Tris-HCl containing 150 mM NaCl and 0.1% Tween 20) three times for 30 min and subsequently probed for 1.5 h with TBS containing heme (10^−6^ M) at room temperature. Nitrocellulose was washed three times for 30 min with TBS-Tween 20 (0.1%) at room temperature. Heme was visualized via it intrinsic peroxidase activity [38] using enhanced chemiluminescence (ECL) reagents (Amersham Pharmacia, Piscataway, N.J). Hbp bands were visualized by exposing the blot to autoradiographic film (Labscientific, Livingston, N.J.).

### Antibody Preparation

200 µl of an emulsion containing purified HbpB (10 µg) and ISA 61 VG adjuvant (Seppic) (120 µl), completed with NaCl 0.9%, were inoculated twice (at a 1-month interval) subcutaneously into 4 weeks old C57B6 mice (Charles River) (Ethics Committee Anses/ENVA/UPEC agreement n°:14/06/2011-1). Two weeks after the second inoculation, 200 µl blood samples were collected every 3 weeks via retro-orbital bleeding. Blood samples were centrifuged twice (3,600 rpm 5 min) to obtain serum which was stored at −20°C. Before use, non-specific antibodies were removed by incubating the immune serum with *E. coli* cell extract for 1 h at 4°C and centrifugation for 10 min at 8,000 rpm. The supernatant was then used as serum.

### Protein Analysis by Electrophoresis

Proteins were analyzed by 12% sodium dodecyl sulfate polyacrylamide gel (SDS-PAGE) electrophoresis [39], followed by Coomassie Blue staining.

### Immunoblot Analysis

Sodium dodecyl sulfate/polyacrylamide gel electrophoresis (SDS-PAGE) and immunoblotting for detecting decreased expression of HbpA in *B. henselae* were performed as follows. Tested strain biomass was collected after 5 days of growth on CBA plates, suspended in PBS buffer and centrifuged. Bacterial pellets calculated to contain 40 µg of proteins were suspended in loading buffer and heated for 5 min at 100°C. Proteins were separated by 12% SDS-PAGE and transferred to a nitrocellulose membrane (Hybond-C Extra, GE Healthcare; VWR International S.A.S Le Périgares - Bât B 201, Rue Carnot 94126 Fontenay-sous-Bois cedex) according to Towbin *et al* [37]. Non-specific binding sites were blocked with 5% skim milk in TBS-Tween 20(0.05%). The immunoblot was probed with polyclonal mouse sera raised against recombinant HbpB (1∶200), followed by a 1∶1,000 dilution of a rabbit anti-mouse IgG horseradish peroxidase[HRP]-conjugated secondary antibody (cell signaling; Ozyme, 10 avenue Ampère, BP 128, 78053 St Quentin en Yvelines France). Visualization of the HRP signal was performed using enhanced chemiluminescence (ECL) reagents (Amersham Pharmacia, Piscataway, N.J). HbpA bands were visualized by exposing the blot to autoradiographic film (Labscientific, Livingston, NJ, USA).

### Protein Assay

The concentration of the protein was determined using the BC assay protein quantitation kit (Interchim).

### Statistical Analysis

Statistical analysis was performed using GraphPad Prism 5 software for Windows. Statistical significance of the data was ascertained by use of Student’s *t* test. A value of P<0.05 was considered significant.

## Results

### Hbps are Able to Bind Congo Red when Expressed in *E. coli*

**Figure 1:**
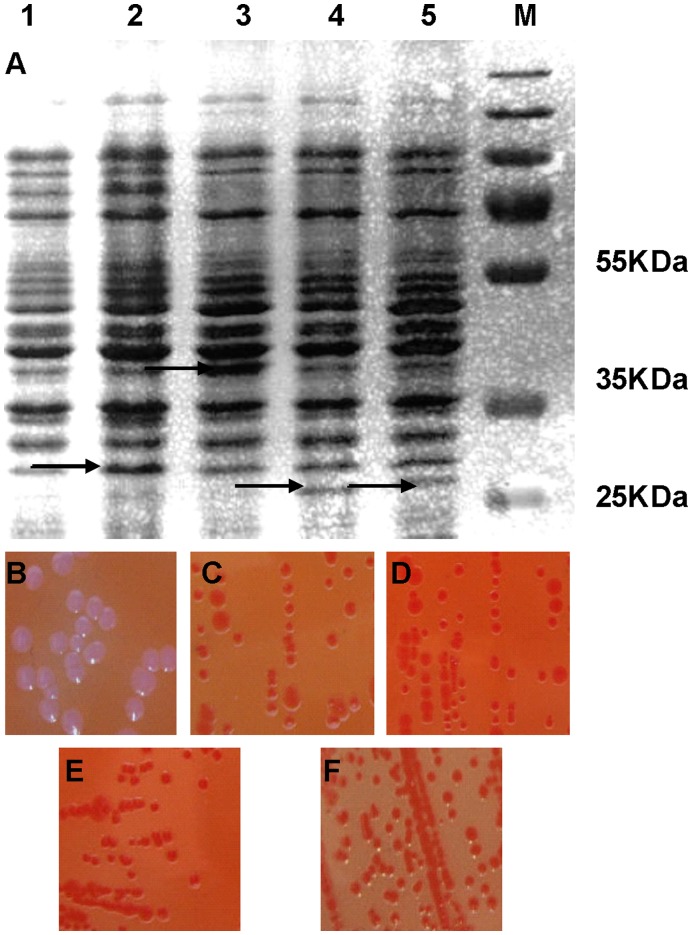
Congo red binding assays. Strains XL1-Blue pBAD24 and XL1-Blue pBAD24 expressing Hbps were expressed for 2 h at 37°C as described in Materials and methods. Expression was evident in SDS-PAGE (A). Line 1: XL1-Blue pBAD24, lines 2 to 5: XL1-Blue pBAD24 expressing HbpA, B, C, D, respectively (20 µg). After plating on Congo red plates supplemented with 0.2% arabinose, strains were grown for 20 h at 37°C. Strain XL1-Blue pBAD24 (B) formed white colonies. Strain XL1-Blue pBAD24*::hbpA* (C), XL1-Blue pBAD24*::hbpB* (D), XL1-Blue pBAD24*::hbpC* (E) and XL1-Blue pBAD24*::hbpD* (F) formed red colonies.

To determine that Hbps of *B. henselae* can bind heme, we first checked for Congo red binding activity, since it had been shown that Hbps can also bind Congo red [40]. Moreover, it had already been shown that expressing a Congo red binding protein on the outer membrane of *E. coli* conferred a Congo red binding phenotype [41]. We transformed *E. coli* XL1-Blue with plasmids pBAD24, pBAD24*::hbpA*, pBAD24*::hbpB,* pBAD24*::hbpC* and pBAD24*::hbpD* to determine whether *hbp* genes can produce a Congo red binding phenotype. Strain XL1-Blue with plasmid pBAD24 did not confer a Congo red phenotype when grown on the plate with 0.2% arabinose (Fig. lB). However, strains expressing HbpA, B, C and D formed a red colony on the plate with 0.2% arabinose (Fig. 1 C, D, E, F). Without arabinose, neither XL1-Blue pBAD24 nor XL1-Blue pBAD24*::hbpA*, XL1-Blue pBAD24*::hbpB*, XL1-Blue pBAD24*::hbpC* or XL1-Blue pBAD24*::hbpD,* conferred a Congo red binding phenotype (data not shown). Taken together, these data showed that Hbps can bind Congo red and confirmed that Hbps were exposed on the cell surface when expressed in *E. coli*.

### Recombinant HbpA, HbpB, HbpC and HbpD can Specifically Bind Heme in vitro

To produce and purify recombinant HbpA, B, C, D from *E. coli*, we amplified their structural gene using *B. henselae* chromosomal DNA as template and primers allowing addition of a six His-tag at the C-terminal of the protein. The fragments were cloned into the pBAD24 plasmid as described in Materials and methods. Plasmids pBAD24*::hbpA,* pBAD24*::hbpB,* pBAD24*::hbpC* and pBAD24*::hbpD* were introduced into strain XL1-Blue. To check for quantities of Hbps in *E. coli* strain XL1-Blue, SDS gel electrophoresis (SDS-PAGE) was used to compare protein extracts of strain XL1-Blue pBAD24::*hbps*_his_ and XL1-Blue pBAD24. A supplementary visible band was observed on SDS page gel for XL1-Blue derivatives harboring plasmid pBAD24*::hbpA,* pBAD24*::hbpB,* pBAD24*::hbpC* or pBAD24*::hbpD* when grown in the presence of 0.4% arabinose (Fig. 1A). Recombinant Hbp proteins were purified by Ni-agarose affinity chromatography as described in Materials and methods. The size of purified Hbp proteins corresponded well to those predicted from their sequences.

**Figure 2:**
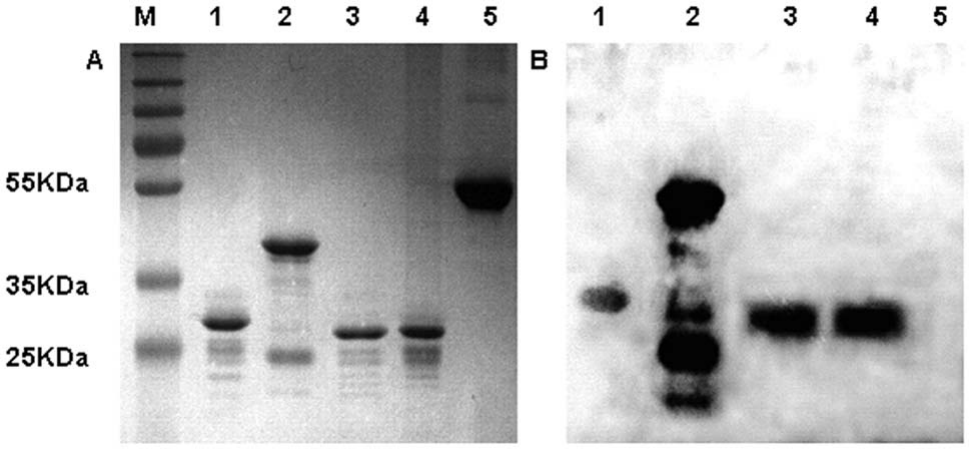
Detection of heme binding by Hbps. After SDS gel electrophoresis, one gel was stained with Coomassie brilliant Blue R. Another gel was transferred to a nitrocellulose filter to perform heme blotting and was detected by ECL as described in Materials and methods. (A) Coomassie Blue staining result. Line 1∶1 µg HbpA, line 2∶1 µg HbpB, line 3∶1 µg HbpC, line 4∶1 µg HbpD, line 5∶3 µg BSA. (B) Heme binding results. Line 1∶1 µg HbpA, line 2∶1 µg HbpB, line 3∶1 µg HbpC, line 4∶1 µg HbpD, line 5∶3 µg BSA.

To test whether pure Hbps can specifically bind heme *in vitro*, a standard method already used for detecting heme binding of cytochrome C was used [36]. Pure HbpA, HbpB, HbpC, HbpD and BSA were separated on two SDS-polyacrylamide gels. One gel was then stained with Coomassie brilliant Blue R (Fig. 2A). Another gel was transferred to a nitrocellulose filter to perform heme blotting and ECL detection (Fig. 2B). Pure Hbps were able to bind heme added at 10^−6^M concentration (Fig. 2B). In contrast, under our assay conditions, BSA was unable to bind heme (Fig. 2B). The latter result underlines the specificity of the proteins that bind heme. Taken as a whole, we conclude that when purified, all Hbps can bind heme *in vitro*.

### Hbps Increase the Efficiency of Heme Uptake Mediated by HemR from *Serratia marcescens* in *E. coli*

Conflicting results concerning the heme transport activity of HbpA encouraged us to first check for the ability of Hbps to transport heme using *E. coli hemA* mutant complementation. Our results demonstrated that Hbps from *B. henselae* did not act as heme transporters (Table 3). These results are at variance with those obtained for Pap31 (HbpA) from *B. henselae* [21], but are in agreement with those obtained for HbpA of *B. quintana* [9] and *Bartonella birtlesii* (Biville F, unpublished data). Heme binding proteins do not act as heme porins, but their ability to bind heme can increase heme concentration on the bacterial surface. This local increase of heme concentration can facilitate its uptake by the bacteria. We checked whether Hbps could modulate the efficiency of the heme uptake process. We first verified the effect of Hbps on the activity of HutA of *B. henselae* expressed in *E. coli*. HutA from *B. quintana* was shown to transport heme and, consequently, to restore growth of an *E. coli hemA* mutant. [9]. Experiments were performed in the presence of high heme concentrations in liquid medium. The weak growth restoration may have been the consequence of mutations enhancing outer membrane permeability [42]. These complementation assays failed for an *E. coli* K-12 *hemA* mutant expressing HutA of *B. birtlessii* when grown on heme-supplemented solid medium [43]. To avoid factual results concerning the heme transportation activity of HutA from *B. henselae*, we expressed it in an *E. coli entF* mutant that cannot grow in the presence of an iron chelator [44]. When a heme transporter is expressed in such strain, addition of heme to the medium restores growth. In order to be used as an iron source, the amount of heme required is 100 times greater than that required for its use as a heme source. Such a complementation assay had already been used to check the heme transport activity of HasR from *Serratia marcescens* [45] and also for characterization of heme-degrading enzymes [46], [47]. No growth was observed in the *E. coli entF* strain expressing HutA from *B. henselae* when grown in iron-depleted medium in the presence of hemoglobin (data not shown). To check for the effect of Hbps from *B. henselae* upon the heme uptake process, plasmids harboring *hbp*s genes were introduced into the *E. coli entF* mutant [44] expressing a HemR heme transporter from *S. marcescens* [48]. Strains obtained were tested for growth on iron-depleted medium in the presence of hemoglobin added at different concentrations. As seen in table 3, HemR alone led to growth around the well containing hemoglobin at 50 µM in the presence of 80 µM dip. Strains expressing *hbpA*, *hbpB*, *hbpC* and *hbpD* were able to grow at lower concentrations of Hb (Table 3), suggesting that HbpA, HbpB, HbpC and HbpD increase the efficiency of the HemR-mediated heme uptake process. HbpB and HbpD were more efficient than HbpA and HbpC (Table 3). To further investigate the efficiency of heme uptake mediated by Hbps and HemR, we grew the bacteria on a minimal iron-depleted plate with 100 µM Dip in the presence of different concentrations of Hb. For the control strain only expressing HemR and strains co-expressing HbpA or HbpC and HemR, no growth was observed around the well whatever the concentration of Hb added (data not shown). The strain co-expressing HbpD and HemR showed growth only around the well containing 50 µM Hb. The strain co-expressing HbpB and HemR was able to grow around the well whatever the concentration of Hb (data not shown). Taken together, we concluded that HbpB and HbpD were more efficient than others at increasing heme uptake when expressed in *E. coli,* and thus they might play an important role at low heme concentrations. Similar results were obtained by introducing plasmids harboring *hbp*s genes in an *E. coli entF* mutant expressing HasR from *S. marcescens* (data not shown). Binding of heme by Hbps increased its concentration around the bacteria and consequently facilitated its uptake.

**Table 3:** The effect of Hbps on HemR-dependent heme uptake.

E. coli strain	Diameter of the halo (cm)			
	50 µM	10 µM	5 µM	1 µM
FB8.27 pBAD24	NM	NM	NM	NM
FB8.27 pBAD24::*hbpA*,B, C, D	NM	NM	NM	NM
FB8.27 pAM::*hemR*,pBAD24	1.82±0.015	NM	NM	NM
FB8.27 pAM::*hemR*,pBAD24::*hbpA*	2.23±0.04	1.78±0.03	1.48±0.09	NM
FB8.27 pAM::*hemR*,pBAD24::*hbpB*	2.77±0.11	2.37±0.08	2.31±0.09	1.84±0.1
FB8.27 pAM::*hemR*,pBAD24::*hbpC*	2.09±0.04	1.64±0.03	1.42±0.08	NM
FB8.27 pAM::*hemR*,pBAD24::*hbpD*	2.55±0.10	1.92±0.09	1.68±0.09	1.22±0.06

*E. coli* strains FB8.27 pBAD24, FB8.27 pBAD24*::hbpA*, FB8.27 pBAD24*::hbpB,* FB8.27 pBAD24*::hbpC*, FB8.27 pBAD24*::hbpD*, FB8.27 pAM239*::hemR* pBAD24, FB8.27 pAM239*::hemR* pBAD24*::hbpA*, FB8.27 pAM239*::hemR* pBAD24*::hbpB,* FB8.27 pAM239*::hemR* pBAD24*::hbpC* and FB8.27 pAM239*::hemR* pBAD24*::hbpD* were tested for efficiency of heme utilization as an iron source in iron-depleted medium M63 (Gly 0.4%, Ara 0.02%, Dip 80 µM, Spc, Amp).

Growth around the wells containing 1 µM, 5 µM, 10 µM, or 50 µM Hb was as described in Materials and methods. After 48 h of growth, the diameter of the zone of turbidity aground the well was measured in quadruplicate for each plate and the mean diameter were calculated. Results are expressed as mean ± SD of the diameter (in cm) obtained for the three plates. NM: Not measurable.

### Hbp Activity is Important for *B. henselae* Growth

**Figure 3:**
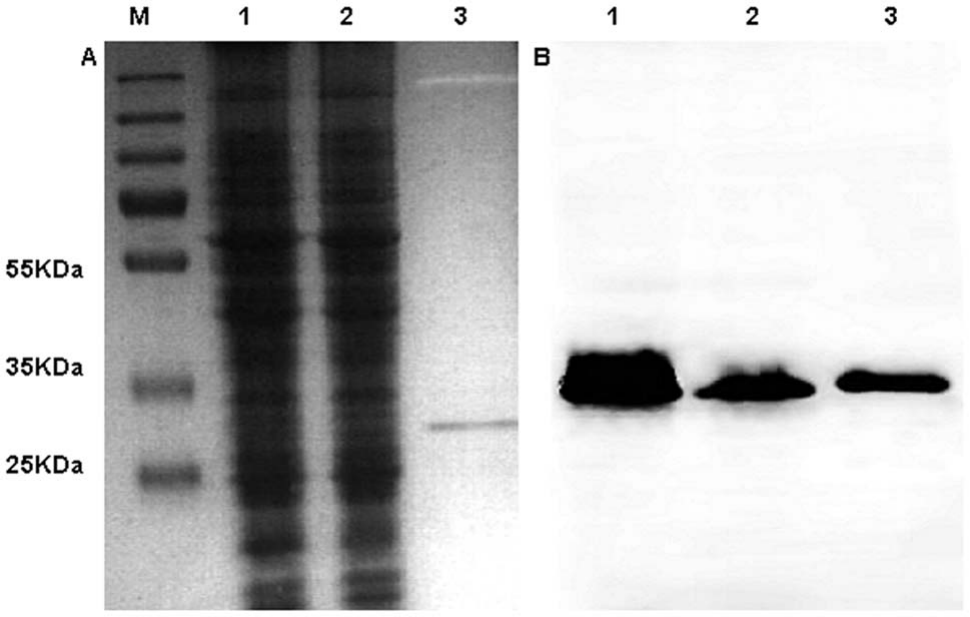
Detection of HbpA expression levels in *B. henselae* pNS2Trc and *B. henselae* pNS2Trc::*hbpA _AS_* by immunoblotting. 40 µg samples of *B. henselae* pNS2Trc (line 1) and *B. henselae* pNS2Trc::*hbpA_AS_* (line 2) and a 100 ng sample of purified his-tagged HbpA (line 3) were loaded on SDS-PAGE. After electrophoresis, one gel was stained with Coomassie brilliant Blue R (A). Another gel was transferred to a nitrocellulose filter for performing immune blotting as described in Materials and methods (B). Measurement of HbpA band intensity using Image J software gave the following results: *B. henselae* pNS2Trc: integrated density: 52; *B. henselae* pNS2Trc::*hbpA_AS_*: integrated density: 26.

Since Hbps can modulate heme uptake efficiency when expressed in *E. coli*, a similar activity was hypothesized in *B. henselae*. In that bacterium, heme serves both as heme and as an iron source [47]. Thus, abolishing or decreasing synthesis of the different Hbps in *B. henselae* could potentially affect its growth capacity. Knockout of HbpA in *B. henselae* was hypothesized as being lethal to the bacteria [20]. A preliminary unsuccessful assay in our lab to disrupt *hbpA* of *B. henselae* was in agreement with this hypothesis. To investigate the function of the four Hbps of *B. henselae* using the same genetic tool, we chose the knockdown method that had been successfully used for *B. henselae* [47], [49], [50]. We cloned *hbps* of *B. henselae* oriented in the reverse direction such that the anti-sense strand was transcribed in plasmid pNSTrc [49]. Plasmids pNS2Trc, pNS2Trc*::hbpA_AS,_* pNS2Trc*::hbpB_AS_* pNS2Trc*::hbpC_AS_* and pNS2Trc*::hbpD_AS_* were introduced into *B. henselae* using electroporation. We first checked for the knockdown effect on the Hbp expression level in *B. henselae* using a western blot experiment. Multiple sequence alignment of Hbps of *B. henselae* revealed a high degree of amino acid sequence conservation at its N-terminal and C-terminal parts even for HbpB, which is longer than other Hbps. According to its size, HbpB can easily be distinguished from other Hbps. Mouse anti-serum against HbpB was prepared and tested for recognition of purified Hbps. Our results demonstrated that anti-HbpB antibody can recognize well-purified HbpB and HbpA. For HbpD and HbpC, recognition by anti-HbpB antibodies was lower or absent (data not shown). Detection of HbpB expression in *B. henselae* was investigated using anti-HbpB anti-serum. We failed to detect HbpB in *B. henselae* using the western blot method. This result is in good agreement with those of proteomic analysis of outer membrane fractions of *B. henselae* and *B. quintana* [51], [52], [53], [54], [55], [56], which showed that HbpB was not detectable using this method. Since HbpA is the most abundant Hbp in *B. henselae* [52], [51], we checked its level in *B. henselae* pNS2Trc and *B. henselae* pNS2Trc*::hbpA*_AS_ using mouse antibody directed against HbpB. As seen in Fig. 3, the level of HbpA was lower in strain *B. henselae* pNS2Trc*::hbpA_AS_* than in strain *B. henselae* pNS2Trc.

**Figure 4:**
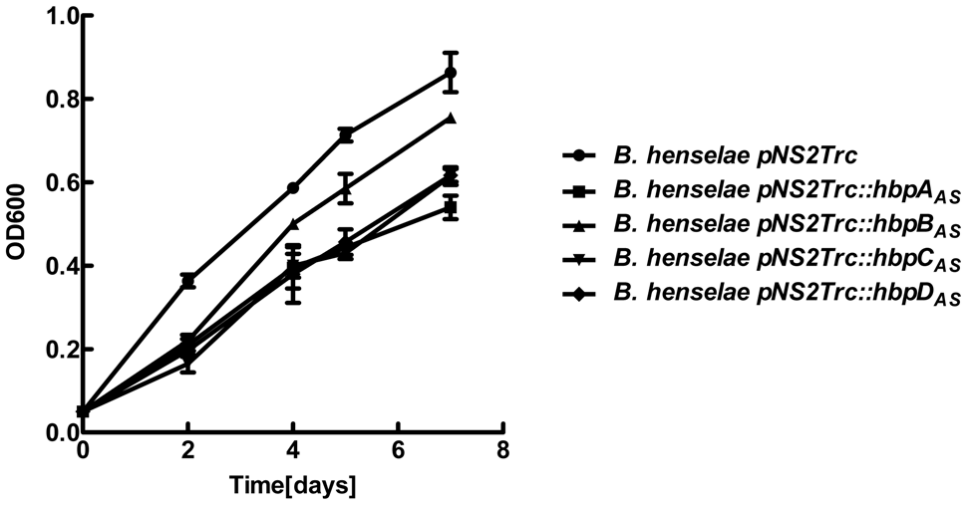
Effect of *hbps* knockdown on growth of *B. henselae* in Schneider’s medium. Strains *B. henselae* pNS2Trc and *B. henselae* pNS2Trc*::hbps_AS_* were cultured in Schneider’s medium, collected after 5-day growth on CBA blood plates and suspended in Schneider’s medium. The bacterial suspension was used to inoculate Schneider’s medium at an OD_600_ of 0.05. Cultures were grown at 35°C in the presence of 5% CO_2_ and OD_600_ was measured on days 2, 4, 5 and 7 after inoculation. All experiments were repeated three times.

The strains obtained were tested for growth on both blood agar plates and in Schneider’s medium as described in Materials and methods. After 6 and 10 days of growth on blood agar plates, colonies produced by strain *B. henselae* harboring pNS2Trc*::hbpA*_AS_, pNS2Trc*::hbpB*_AS_, pNS2Trc*::hbpC*_AS_ or pNS2Trc*::hbpD*_AS_ were much smaller than those formed by *B. henselae* pNS2Trc (Table 4). The *B. henselae* strain containing pNS2Trc::*hbps_AS_* also grew more slowly than *B. henselae* pNS2Trc in Schneider’s medium (Fig. 4). These results indicated that a decrease in the amount of Hbps slowed growth of *B. henselae*.

**Table 4:** Effect of *hbps* knockdown on growth of *B. henselae* on blood plates.

Strain	Colony size (mm)	
	Day 6	Day 10
***B. henselae*** (pNS2Trc)	**0.58±0.12**	**1.00±0.149**
***B. henselae*** (pNS2Trc*::hbpA_AS_*)	**NM**	**0.50±0.105 (p<0.0001)**
***B. henselae*** (pNS2Trc*::hbpB_AS_*)	**NM**	**0.53±0.125 (p<0.0001)**
***B. henselae*** (pNS2Trc*::hbpC_AS_*)	**NM**	**0.51±0.120 (p<0.0001)**
***B. henselae*** (pNS2Trc*::hbpD_AS_*)	**NM**	**0.449±0.072 (p<0.0001)**

For the growth test on CBA plates, strains *B. henselae* (pNS2Trc), *B. henselae* (pNS2Trc*::hbpA _AS_*), *B. henselae* (pNS2Trc*::hbpB _AS_*), *B. henselae* (pNS2Trc*::hbpC _AS_*) and *B. henselae* (pNS2Trc*::hbpD_AS_*) were collected after 5 days of growth on CBA plates and suspended in PBS buffer to obtain about 10^3^ CFU ml^−1^. Two-hundred microliters of cell suspension were plated on the CBA plate. Colony sizes were measured after 6 and 10 days of growth at 35°C in the presence of 5% CO_2_. Data are the mean diameter (mm) ± SD of 10 colonies from one representative experiment. Standard deviations were calculated using Statview software. All experiments were repeated three times. NM: not measurable.

### Knockdown of Hbps Increased *B. henselae* Sensitivity to Hydrogen Peroxide

**Figure 5:**
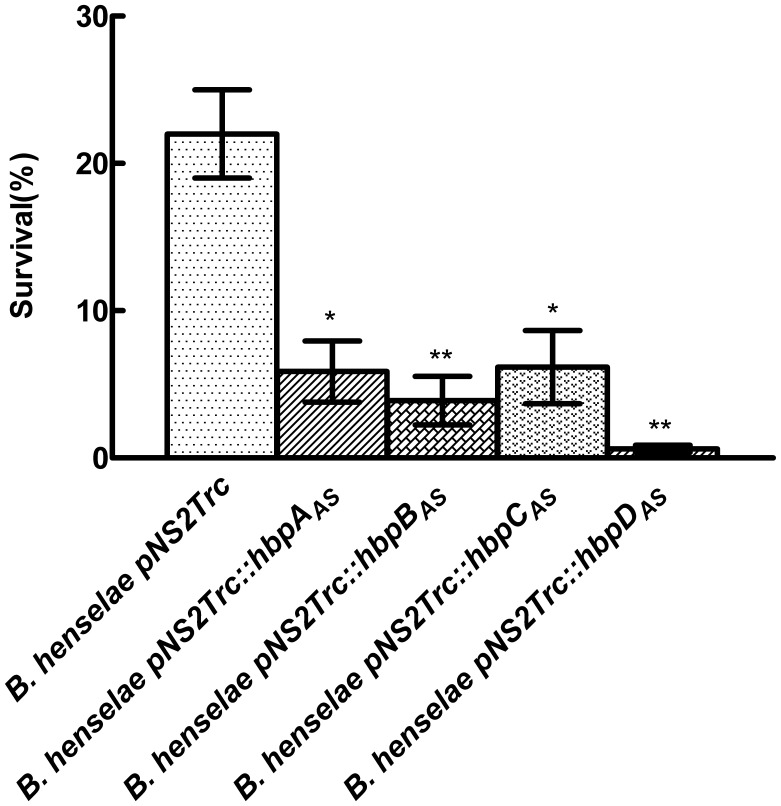
Hbp knockdown decreases the ability of *B. henselae* to undergo exposure to H_2_O_2_. *B. henselae* pNS2Trc and *B. henselae* pNS2Trc::*hbps*_AS_ were challenged with 10 mM H_2_O_2_ as described in Materials and methods. Experiments were performed in triplicate; survival rates were expressed as described in Materials and methods. (*P<0.05, **P<0.01 compared to *B. henselae* pNS2Trc).

Analyses of *Bartonellae* genomes demonstrated that numerous genes involved in the oxidative stress response were not present. However, it was shown that *Bartonella bacilliformis* was able undergo oxidative stress generated by exposure to H_2_O_2_ [15]. It was suggested that one potential role for Hbps was to bind heme at the surface of the bacteria and provide an antioxidant barrier via heme intrinsic peroxidase activity [57]. To examine this hypothesis, we tested the effect of *hbp* knockdown on the ability of *B. henselae* to face 30 min exposure to 1 mM and 10 mM hydrogen peroxide. After exposure to 1 mM H_2_O_2,_ survival was about 50% for both control strain *B. henselae* pNS2Trc and *B. henselae* pNS2Trc*::hbpC_AS._* There existed slightly decreased survival capacity for *B. henselae* pNS2Trc::*hbpA_AS_*, *B. henselae* pNS2Trc::*hbpB_AS_* and *B. henselae* pNS2Trc::*hbpD_AS_* (data not shown). After exposure to 10 mM H_2_O_2_, survival was about 25% for control strain *B. henselae* pNS2Trc. With strain *B. henselae* pNS2Trc::*hbpA_AS_*, *B. henselae* pNS2Trc::*hbpB_AS_* and *B. henselae* pNS2Trc::*hbpC_AS,_* sensitivity to hydrogen peroxide increased about 3-4-fold (Fig. 5). Decreasing HbpD levels more sharply increased *B. henselae* sensitivity to hydrogen peroxide (Fig. 5). These results indicated that lowering the Hbp level in *B. henselae* significantly increased its sensitivity to H_2_O_2_.

### Effect of Hbp Knockdown on *B. henselae* Capacity to Invade Endothelial Cells

Within cells, bacterial infection was shown to induce ROS production [58]. Thus, decreasing the ability to undergo oxidative stress is expected to decrease the ability of *B. henselae* to survive in endothelial cells. This promoted us to check the effect of Hbp knockdown upon the capacity of *B. henselae* to invade human endothelial cells and to survive within them.

**Figure 6:**
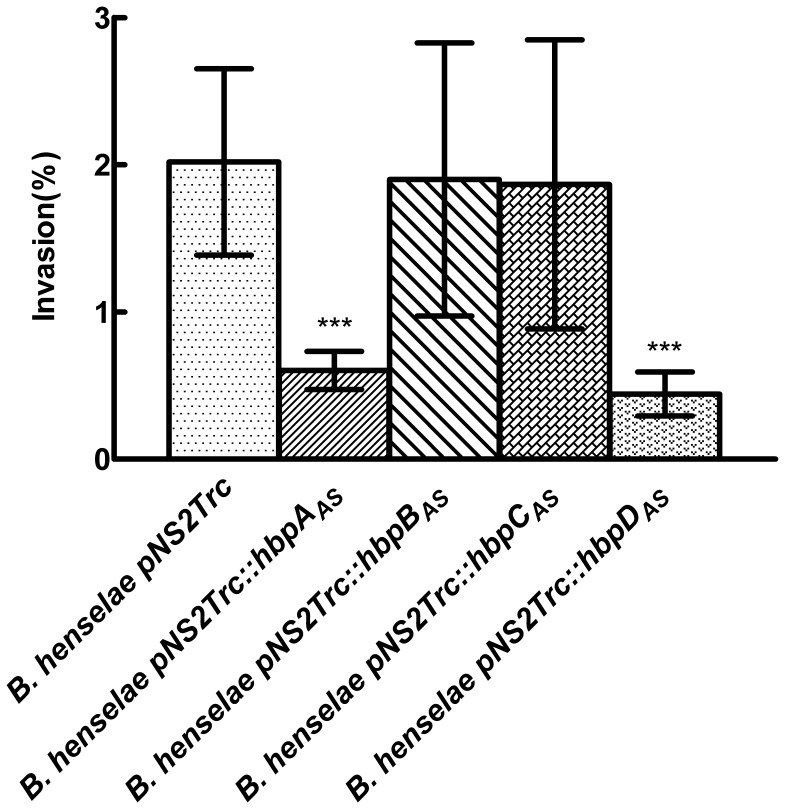
Effect of Hbp knockdown on endothelial cell invasion. Invasion of endothelial cells by *B. henselae* pNS2Trc and *B. henselae* pNS2Trc*::hbps_AS_.* Cells were mixed with bacteria at 0.1 m.o.i. After 24 h, infected cell were treated with gentamicin to kill extracellular bacteria and lysates were plated on the CBA blood plate to determine the number of intracellular bacteria. Invasion was calculated using the equation provided in Materials and methods. (***P<0.005 compared to *B. henselae* pNS2Trc).

For endothelial cell invasion, both *B. henselae* pNS2Trc*::hbpB_AS_* and *B. henselae* pNS2Trc*::hbpC_AS_* exhibited the same invasion rate as control strain *B. henselae* pNS2Trc (about 2%) (Fig. 6). However, invasion rates of *B. henselae* pNS2Trc*::hbpA_AS_* and *B. henselae* pNS2Trc*::hbpD_AS_* decreased 3-fold compared to the control strain (Fig. 6).

**Figure 7:**
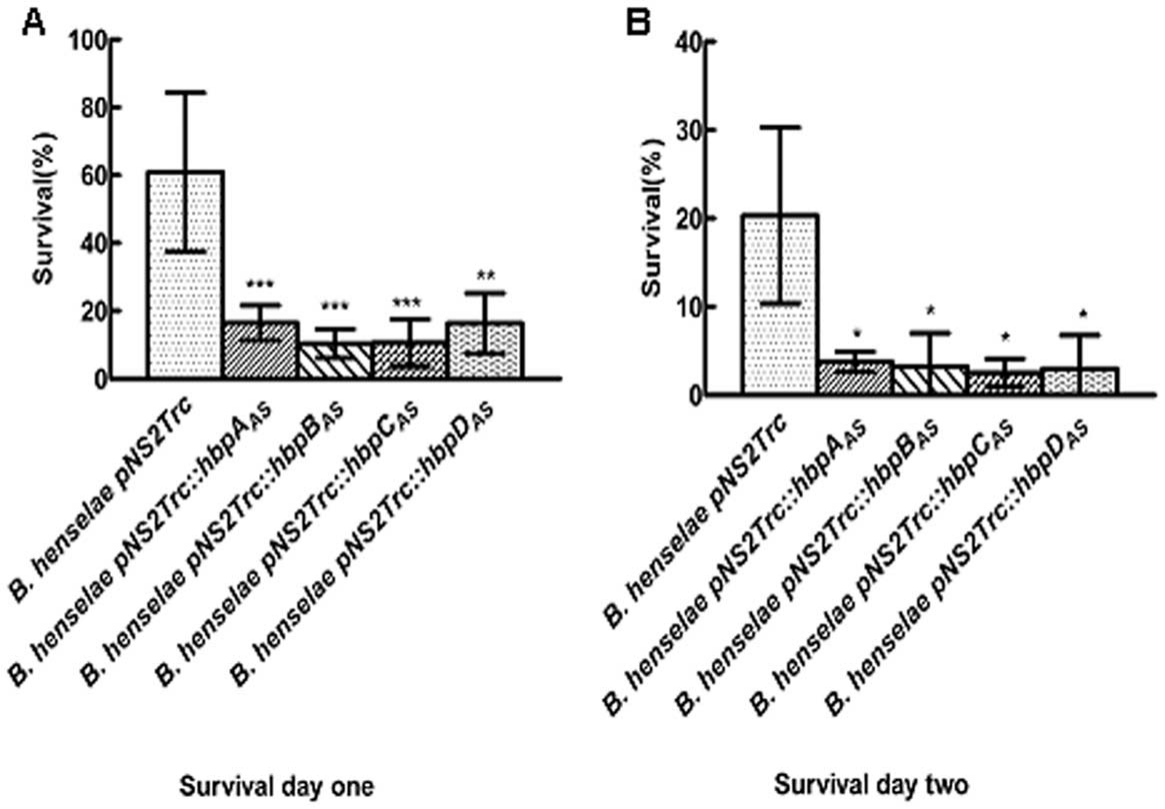
Effect of Hbp knockdown on survival of *B. henselae* in endothelial cells. Survival of *B. henselae* pNS2Trc and *B. henselae* pNS2Trc*::hbps_AS_* in endothelial cell. After gentamicin killing, infected cell were grown for 24 h or 48 h in modified DMEM medium. Lysates were incubated in Schneider’s medium overnight. Then lysates were collected after centrifugation and plated on CBA blood plates to determine the number of intracellular bacteria. The survival rates after 24 h growth (A) and 48 h growth (B) were calculated using the equation provided in Materials and methods. (*P<0.05, **P<0.01, ***P<0.005 compared to *B. henselae* pNS2Trc).

For survival in the endothelial cell assay, mixtures were grown for 24 h or 48 h after the gentamicin killing assay. After 24 h in endothelial cell, cell lysates were spread on the blood plate to check viable bacterial number. Surprisingly, no bacteria were visible on the blood plate after 2-week incubation. To overcome this problem, we first grew the mixture in Schneider’s liquid medium overnight, sustaining primary isolation of *B. henselae* [29] before plating it on blood plates. Colony count after 2-week incubation showed a decrease in viable bacteria for all strains tested, though it has been claimed that *B. henselae* is able to replicate inside endothelial cell through bacterial rRNA replication [59]. Survival rates for *B. henselae* pNS2Trc*::hbps_AS_* were much lower than those of control *B. henselae* pNS2Trc (Fig. 7), after 24 h or 48 h growth in endothelial cells. To check for an effect of overnight growth in Schneider’s medium, about 600 *B. henselae* pNS2Trc or *B. henselae* pNS2Trc*::hbps_AS_* bacteria were grown overnight in that medium and plated on blood plates for enumeration. The increase in bacterial numbers was calculated for all tested strains. The increase was about 20–30% for both *B. henselae* pNS2Trc and *B. henselae* pNS2Trc*::hbps_AS_*. The differing survival rates of *B. henselae* pNS2Trc and *B. henselae* pNS2Trc::*hbp*s *_AS_* were not related to a growth defect in Schneider’s medium. We conclude that Hbps of *B. henselae* play an important role in survival within endothelial cells.

### Hbps are Involved in Multiplication of *B. henselae* in *C. felis*

It was demonstrated that *B. henselae*-infected cat fleas (*C. felis*) can transmit *B. henselae* to cats [60] and that *B. henselae* can replicate in the gut of the cat flea [61]. Inside the arthropod gut, bacteria confront oxidative stress after each blood meal [16]. Since Hbps of *B. henselae* play a protective role against H_2_O_2_-produced oxidative stress, this prompted us to check for an Hbp knockdown effect on *B. henselae* multiplication in fleas. After feeding fleas for 2 days with blood containing the bacteria, fleas were fed with blood without bacteria for another 8 days. For control strain *B. henselae* pNS2Trc, we were able to detect *B. henselae* DNA in the feces from day 1 to day 10 (Table 5). For strains with a decreased amount of HbpA, HbpB, HbpC or HbpD, no bacterial DNA could be detected after day 6. To exclude the possibility that this result was due to small amounts or to the quality of DNA from flea feces, we amplified flea 18S rDNA from day-7-to-10 flea feces samples using primers Cf18Sf and Cf18Sr. Flea 18S rDNA was detected in all of the day-7-to-10 flea feces samples (data not shown). This suggests that Hbps play an important role in multiplication of *B. henselae* in fleas.

**Table 5:** Detection of *B. henselae* DNA from flea feces samples using PCR.

	Flea feces samples (days)									
Strain	1	2	3	4	5	6	7	8	9	10
*B. henselae* pNS2Trc	+	+	+	+	+	+	+	+	+	+
*B. henselae* pNS2Trc*::hbpA_AS_*	+	+	+	+	+	+	−	−	−	−
***B. henselae*** ** pNS2Trc** ***::hbpB_AS_***	+	+	+	+	+	+	−	−	−	−
***B. henselae*** ** pNS2Trc** ***::hbpC_AS_***	+	+	+	+	+	+	−	−	−	−
***B. henselae*** ** pNS2Trc** ***::hbpD_AS_***	+	+	+	+	+	+	−	−	−	−

About 500 fleas were first feed with blood containing 500 µl bacteria (2×10^8^/ml) for 2 days and then fed uninfected blood for 8 days. Flea feces were collected every day. DNA was extracted from flea feces and PCR was performed as described in Materials and methods.

## Discussion

In this report, we investigated the role of *B. henselae* Hbps in heme utilization, the oxidative stress response, cell colonization and survival within arthropod vector *C. felis*. Previous data had shown that recombinant HbpA of *B. quintana* was able to bind heme *in vitro,* but did not confer a heme binding phenotype *in vivo* when expressed in *E. coli* [22]. Later it was claimed that HbpB of *B. quintana* did not bind heme [19]. Recently, it was shown that *hbpC* of *B. henselae,* when expressed in *E. coli,* confers a heme binding phenotype *in vivo* [20].

Our results clearly show that expression of all Hbps from *B. henselae* in *E. coli* confers a Congo red binding phenotype (Fig. 1), thus suggesting that Hbps have a surface location when expressed in *E. coli.* We also demonstrate that, *in vitro*, all purified Hbps specifically bind heme. To characterize the physiological importance of Hbps, we first investigated their effect on the heme uptake process. Pap31 (HbpA) of *B. henselae* was claimed to act as a heme porin when expressed in *E. coli* [21], but conflicting data about this activity were published for HbpA of *B. quintana* [57]. Moreover, we failed to visualize any heme porin activity for Hbps of *B. henselae* when expressed in *E. coli* (data not shown). Based on the above results, we hypothesized that the Hbp family of *Bartonella* could act as a heme reservoir, thus rendering it available under heme-limited conditions. This hypothesis is in good agreement with the absence of genes encoding for heme and iron storage proteins in *Bartonellae* genomes. Such heme storage activity might enhance the efficiency of the heme uptake process. We examined the effect of Hbps on the activity of heterologous heme transporters HemR and HasR of *S. marcescens* [48], [62]. All Hbps increased heme uptake efficiency mediated by HemR and HasR. Efficiency at low heme concentrations was better with HbpB and HbpD than with HbpA and HbpC. Such differences in efficacy could be related to the differing levels of Hbps in *E. coli.* This is the case for HbpB, but not for HbpD (Fig. 1). This result suggests that HbpD might be active when the heme concentration is low. This conclusion is in good agreement with the increase in *hbpD* expression when *B. quintana* was grown in the presence of low heme concentrations [19]. How heme is transferred from heme binding proteins to HemR and HasR heme transporters remains unknown.

During mammal and flea invasion, *Bartonellae* must face microenvironmental shifts, stress and the host immune defense. For example, it was shown that reactive oxygen species (ROS: O_2_^−^, H_2_O_2_ and OH^−^) production is an important immune defense mechanism for mammal hosts and arthropod vectors against pathogenic bacteria [16], [63]. Recently, it was shown that ROS (H_2_O_2_) levels in midgut were higher (over 10 mM) in *Y. pestis* infected fleas. Antioxidant treatment prior to infection decreased ROS levels and resulted in higher *Yersinia pestis* loads [64]. An OxyR *Y. pestis* mutant showed reduced growth in fleas early after infection [64]. ROS are potentially toxic for both the host cell and pathogenic bacteria [16], [65], [66], [67]. Host cells are protected from oxidative damage by enzymes that detoxify ROS, such as SOD, catalase, glutathione peroxidase (Gpx) and thioredoxin peroxidase, that detoxify H_2_O_2_ [68]. In arthropod vectors, the adaptive response to ROS has also been thoroughly investigated [16]. *B. henselae* replicates in the gut of the cat flea and is able to survive several days in flea feces [61]. It was shown that the hematophagous vector has a substantial need for huge amounts of blood at each meal; digestion of hemoglobin within the gut of the vector releases large quantities of heme, which has potential pro-oxidant and cytotoxic effects if not bound to proteins [69], [70]. To successfully replicate in the cat flea gut, *B. henselae* must confront toxic ROS and repair damage. Many homologues of genes involved in the oxidative stress response in *E. coli* are not present in the *B. henselae* genome. The absence of homologues of these genes suggests that *Bartonellae* must possess uncharacterized mechanisms in response to oxidative stress. One actor in this oxidative stress response was recently identified as being a heme-degrading enzyme enabling release of iron from heme [47]. In this report, we demonstrate that all Hbps are required to efficiently undergo exposure to hydrogen peroxide. A role for the intrinsic peroxidase activity of heme bound to Hbps was hypothesized for *B. quintana* [19] and was shown to be the case for *Porphyromonas gingivalis* [71]. The competence of Hbps in detoxifying H_2_O_2_ might also be required for efficient invasion and survival in endothelial cells, major target cell types for bacterial colonization in the reservoir host(s) as well as in the infected host [72]. Bacterial infection was shown to induce ROS production in endothelial cells [58]. As a consequence, the weaker survival capacity in endothelial cells, related to knockdown of all Hbps, might be related to lower resistance to oxidative stress.

For HbpA and HbpD, we also show a weakening in the endothelial cell invasion process when their level decreases. For HbpA, this effect might be related to its Opa domain, which has been shown to be involved in entry into the host epithelial cell in *Neisseria gonorrhoeae* [73]. Indeed, previous data also showed that Pap31 (HbpA) of *B. henselae* was able to bind endothelial cells in a dose-dependent manner, and binding was inhibited by anti-Pap31 antibodies [24]. However, HbpD involvement in the endothelial cell invasion process is striking, and can be explained by greater sensitivity to oxidative stress produced by endothelial cells, thus leading to more rapid killing of bacteria. This hypothesis is supported by the fact that the mutant of HbpD cannot delay lysosomal fusion, leading to significantly reduced viability within endothelial cells [27].

Clearance of bacteria in the flea feces can be explained by a decrease in survival within the arthropod. Our results clearly demonstrate more rapid clearance of *B. henselae* in the flea feces when invasion assays are performed using Hbp knockdown mutants. In addition, this more rapid clearance of bacteria in the flea feces can be attributed to decreased ability to confront oxidative stress. Thus, the ROS-detoxifying activity of Hbps also plays an important role during colonization of fleas. Taken together, we reveal the functions of Hbps in heme utilization, the oxidative stress response, cell colonization and flea transmission. The ability of Hbps to bind heme provides competence in destroying ROS, thus constituting an important immune defense system for host cell and arthropod vectors. Functional identification of Hbp families in cell interactions and flea transmission should help to develop strategies for fighting infection and transmission. It will be interesting in the future to elucidate anti-oxidant mechanisms used by other vector-borne pathogens.
